# An Improved Method for Measuring Acoustic Attenuation in Viscoelastic Solid Media

**DOI:** 10.3390/mi17070869

**Published:** 2026-07-22

**Authors:** Lin Fa, Jinyue Li, Huiting Yang, Yulin Xu, Hongyi Zhu, Xiangrong Fang, Xiao Zou, Ning Shen, Meishan Zhao

**Affiliations:** 1 College of Science and Engineering, Xi’an Fanyi University, Xi’an 710105, China; 2 School of Electronic Engineering, Xi’an University of Posts and Telecommunications, Xi’an 710121, China; 17791275024@163.com (J.L.); 18996018501@163.com (Y.X.); 13223230768@163.com (H.Z.); fangxr@xupt.edu.cn (X.F.); shenning@xupt.edu.cn (N.S.); 3 Lhasa Branch, China Telecom Co., Ltd., Lhasa 850000, China; 18334982702@163.com; 4 Well Logging Technology Research Institute, China National Petroleum Corporation Well Logging Co., Ltd., Xi’an 710077, China; szzoux@cnpc.com.cn; 5 James Franck Institute and Department of Chemistry, The University of Chicago, Chicago, IL 60637, USA

**Keywords:** acoustic attenuation, longitudinal waves, damping attenuation, wave propagation attenuation, particle vibration, internal stress

## Abstract

Accurate measurement of attenuation during acoustic wave propagation in viscoelastic solid media is of theoretical and practical significance. Conventional studies primarily rely on analogies to models of electromagnetic wave attenuation in non-ideal media. Many modern models of acoustic attenuation rely on continuum mechanics and complex material properties. Although there are similarities between acoustic and electromagnetic waves, conventional models overlook their fundamental physical differences and neglect the influence of particle-vibration damping in viscoelastic media. Additionally, in applications with a single-transmitter and dual-receiver configuration, the effects of specific characteristics on the measurement of the acoustic attenuation coefficient are eliminated in both the electric–acoustic conversion of the transmitting transducer and the acoustic–electric conversion of the receiving transducer. The discrepancies in geometric parameters (size and shape) between the two measurement modules lead to inconsistent frequency responses, thereby introducing measurement errors in acoustic attenuation. To address these issues, we investigate the coupling mechanism between particle vibration damping and wave propagation attenuation, derive an analytical expression for the acoustic attenuation coefficient that accounts for this coupling, and propose a new method for accurately measuring acoustic attenuation in viscoelastic solid media. The experimental results validate the theoretical predictions of acoustic attenuation.

## 1. Introduction

Acoustic waves involve particle vibration in a medium, resulting in wave propagation. Particle vibration damping and wave-propagation attenuation are two fundamental physical concepts that describe energy loss of acoustic waves in the medium. Vibration damping is the phenomenon in which the amplitude of particle vibration decreases over time, mainly due to dissipative factors such as internal friction and viscous resistance within the medium, and it reflects the time-domain energy dissipation. In propagation attenuation, the amplitude of a wave gradually decreases due to energy dissipation arising from the medium’s viscoelastic properties, which is reflected in the spatial domain. In nature, media have certain viscosity and thermal conductivity, whether in fluids or solids, which cause damping attenuation of particle vibration in the time domain and attenuation of wave propagation in the spatial domain. So, the attenuation of acoustic wave propagation in viscoelastic media is composed of the damping of particle vibration in the time domain and the attenuation of wave propagation in the spatial domain.

Several major factors affect acoustic attenuation in media, including viscosity, heat conduction, acoustic scattering by tiny particles (or pores) within the medium, and geometric diffusion. When the pore size in the medium is much smaller than the wavelength, the effect of acoustic scattering on acoustic attenuation is negligible. In petroleum exploration, the wavelengths of seismic and acoustic logging signals are usually much larger than the tiny particles and pores within dense, continuous rock layers, so their effects on acoustic attenuation can be disregarded.

Many researchers have conducted extensive theoretical and experimental studies from both macroscopic and microscopic perspectives in understanding the mechanism of acoustic attenuation. Attenborough [1], Qian [2], and Challis [3] used the dense-suspension acoustic wave attenuation model to investigate the influence of pore-induced acoustic scattering on acoustic attenuation. Considering viscosity and the presence of pore particles in rock layers, Babick and Richter analyzed the influence of the coupling between viscosity and inertia [4]. Verma et al. [5] investigated the influence of thermal conductivity. For many underground rock formations, thermal conductivity is low, leading to minimal acoustic energy dissipation from temperature gradients. So, this factor may be ignored. In the far-field region, acoustic waves can be approximately treated as plane waves (neglecting geometric attenuation). Under these conditions, acoustic attenuation is mainly due to the medium’s viscosity.

Geophysicists also studied acoustic attenuation caused by complex underground geological structures. Yang [6], Long [7], Hefner [8], and Jiang [9] experimentally studied acoustic attenuation in seafloor sediments; Zheng et al. [10] investigated acoustic attenuation in gassy sediments; Wang et al. [11] experimentally studied acoustic speed and attenuation in gas-bearing sediments; Meyer et al. [12] examined glacier attenuation in the frequency range of 2–35 kHz; Cooper [13] studied energy loss and attenuation of seismic waves propagating in viscoelastic rocks; Zou et al. [14] proposed a method to calculate the attenuation of head waves from acoustic signals propagating in seafloor sediments; Zimmer et al. [15] experimentally measured the frequency-dependent relationship of acoustic attenuation coefficients in seafloor sands and gravels over the frequency range of 1 kHz to 400 kHz; Wan et al. [16] measured the attenuation of long-distance broadband acoustic signals under seabed conditions.

The study and measurement of acoustic attenuation mentioned above rarely accounted for the influence of particle vibration damping on acoustic attenuation, nor did they consider the effect of the electrical–acoustic/acoustic–electrical conversion of the acoustic transducers on the characteristics of the measured acoustic wave signals. In the following discussion, we refer to particles, micro-particles, and minute-volume elements as “particles”. In a non-ideal medium with nonzero electrical conductivity, electromagnetic waves possess energy but no mass. There is no transient transition process or damping attenuation at any spatial point; only spatial-domain propagation attenuation occurs. Part of the energy radiated by the acoustic source is used to compensate for thermal losses caused by propagation attenuation, and the remaining energy provides the energy for the forward propagation of the electromagnetic wave.

Auld [17] employed an analogy between acoustic and electromagnetic waves to study wave attenuation during propagation in viscoelastic solids and derived an analytical expression for the acoustic attenuation coefficient. However, that expression does not account for the effect of particle vibration damping on the acoustic attenuation coefficient. In reality, vibrating particles within a viscoelastic medium possess energy, mass, and inertia. Therefore, for acoustic waves propagating in a viscoelastic medium, there exists both particle vibration damping in the time domain and wave propagation attenuation in the spatial domain.

Part of the energy radiated by the acoustic source is used to compensate for thermal loss caused by particle vibration damping; another part compensates for thermal loss caused by wave propagation attenuation; and the remaining part provides the energy for the forward propagation of the acoustic wave. Fa and Zhao et al. [18,19] reported the difference between the acoustic attenuation coefficient of viscoelastic media and the electromagnetic wave attenuation coefficient of non-ideal media and attempted to conduct a theoretical derivation for this phenomenon. Although progress was made, their analytical expression was still incomplete and failed to comprehensively describe the attenuation mechanism of acoustic waves in viscoelastic media from a physical perspective. Based on the principle of energy conservation, the coupling mechanism between particle vibration damping and wave attenuation was explored, and the physical basis of acoustic attenuation was thoroughly explained.

From the constitutive relationship of viscoelastic media, a new analytical expression for the acoustic attenuation coefficient was derived [19]. This expression accounts for both the damping effect of particle vibration in the time domain and the attenuation of wave propagation in the spatial domain. Furthermore, based on previous reports on the characteristics of piezoelectric transducers [20,21], an improved method for measuring the acoustic attenuation coefficient was proposed, effectively eliminating the influence of the transducer’s electric–acoustic/acoustic–electric conversion characteristics on the measurement results. Both numerical calculations and experimental verification indicate that the proposed analytical expression for acoustic attenuation is highly accurate and reliable, and that the developed measurement method is also effective.

## 2. Physics Model

If energy losses from acoustic scattering and thermal conduction are neglected, the essence of acoustic attenuation stems from thermal losses due to the frictional resistance force acting on the particles. Damping attenuation is the physical phenomenon in which the amplitude of particle vibration decreases over time. It is mainly caused by dissipative factors, such as internal frictional resistance force within the medium, and it reflects the time-decay characteristic of the vibration system. Propagation attenuation reduces the amplitude of those particles due to viscous resistance, resulting in a decrease in vibrational amplitude with increasing propagation distance, reflecting the wave’s spatial decay.

An acoustic source continuously radiates sine waves of a fixed frequency into the surrounding medium. Along the propagation path of the acoustic wave, each particle within the medium undergoes a transient process from rest to steady-state sine vibration. After all particles in the region of interest have entered the steady-state sine vibration state, the acoustic energy continuously radiated by the source exactly compensates for the energy lost due to thermal dissipation caused by frictional resistance acting on the particles within the medium, thereby maintaining their steady-state sine vibration.

From the surface phenomenon, at this time, only wave-propagation attenuation in the spatial domain occurs. However, in essence, this is the actual attenuation caused by the propagation of acoustic waves in a viscoelastic medium. This attenuation is composed of particle-vibration damping and wave-propagation attenuation due to energy dissipation during wave propagation and is called single-frequency acoustic attenuation. The energy radiated by a single-frequency sine acoustic source outward is partly used to compensate for the energy loss caused by particle vibration damping, another part is used to compensate for the heat loss caused by wave propagation attenuation, and the remaining part is used to provide the energy required for the forward propagation of the acoustic wave.

### 2.1. Related Works

According to reference [21], the impulse response and system function of the internal vibrating particle within the viscoelastic solid can be expressed as

(1) $$h(t)=Ae^{-\beta t}\cos(\omega_{d}t+\theta )\varepsilon (t)$$

(2) $$H(\omega )=H(s){|}_{s=i\omega }=\frac{i\omega C_{m}}{-mC_{m}\omega^{2}+iR_{m}C_{m}\omega +1}$$

(3) $$\beta =\frac{R_{m}}{2m}$$

(4) $$\omega_{d}=\frac{\sqrt{4mC_{m}-{\left(R_{m}C_{m}\right)}^{2}}}{2mC_{m}}$$

The variables in Equations (1)–(4) are the imaginary unit (i), the unit step function (ε(t)), the parameter describing the vibration damping (β) of the particle under the influence of only spring force and viscous resistance, the angular frequency of the damping vibration ($\omega_{d}$), the amplitude (A), and the initial phase angle (θ). Equations (1) and (2) indicate that the physical parameters of the viscoelastic solid medium determine the mechanical impulse response and system function of the particle vibration system. We note that the initial displacement of the particles within the viscoelastic medium is nonzero (i.e., the initial state is nonzero). It is subjected only to viscous resistance and elastic force, resulting in damped oscillation (i.e., zero-input response), with the damping attenuation coefficient given by Equation (3).

Vibrating particles in a viscoelastic solid cause adjacent particles to vibrate via internal stress, and this process repeats sequentially, leading to the propagation of acoustic waves through the medium. Based on references [17,21], the propagation attenuation coefficient, wave number, and phase velocity of the longitudinal wave can be expressed by

(5) $$\alpha_{1}=\omega {\left(\frac{\rho }{2c_{11}}\right)}^{1/2}\left\{\frac{1}{{\left[1+{\left(\omega \eta_{11}/c_{11}\right)}^{2}\right]}^{1/2}}-\frac{1}{1+{\left(\omega \eta_{11}/c_{11}\right)}^{2}}\right\},$$

(6) $$k=\sqrt{\frac{\rho }{2c_{11}}}\frac{\omega^{2}\eta_{11}/c_{11}}{1+{\left(\omega \eta_{11}/c_{11}\right)}^{2}}{\left\{\frac{1}{{\left[1+{\left(\omega \eta_{11}/c_{11}\right)}^{2}\right]}^{1/2}}-\frac{1}{1+{\left(\omega \eta_{11}/c_{11}\right)}^{2}}\right\}}^{-1/2}$$

and

(7) $$v_{p}=\frac{\omega }{k}={\left(\frac{2c_{11}}{\rho }\right)}^{1/2}\frac{1+{\left(\omega \eta_{11}/c_{11}\right)}^{2}}{\omega \eta_{11}/c_{11}}\left\{\frac{1}{{\left[1+{\left(\omega \eta_{11}/c_{11}\right)}^{2}\right]}^{1/2}}-\frac{1}{1+{\left(\omega \eta_{11}/c_{11}\right)}^{2}}\right\},$$

respectively.

### 2.2. Single-Frequency Acoustic Attenuation

According to the law of conservation of energy, when the sine force acting on particles within a viscoelastic medium suddenly ceases, the energy dissipated during the subsequent damped oscillation, i.e., the transient process from steady-state sine vibration to static state, should equal the energy previously supplied by the continuous sine force that had driven the particles into steady-state sine vibration. Thus, we derived a new sine steady-state acoustic attenuation coefficient that accounts for both the damping attenuation of particle vibration in the time domain and the attenuation of wave propagation in the space domain. The sine force acting on the particles within the viscoelastic medium must counteract the frictional resistance force caused by viscosity to enable the particles to maintain a stable sine vibration state.

If the time required for a sine longitudinal wave to propagate from the origin of the coordinate system to the spatial position *x* in a viscoelastic solid is *t*, then, based on Equations (3) and (5), the displacement of the particle at the spatial position *x* where the acoustic wave reaches at time *t* can be expressed as

(8) $$u=e_{x}Ae^{-\beta t}e^{-\alpha_{1}x}e^{i(\omega t-kx+\theta )}$$

When the sine force acting on the particle is suddenly removed, the system enters a damping oscillation process. The energy consumed during this process is equal to the energy input when the continuous sine force previously forced the particle to perform steady-state sine vibration. Based on this energy equivalence relationship, the damping attenuation characteristic of the particle in the time domain can be equivalently regarded as an additional term in the attenuation of the wave propagation in the spatial domain (i.e., $e^{-\beta t}=e^{-\beta x/v_{p}}=e^{-\alpha_{e}x}$). Therefore, Equation (8) can be rewritten as

(9) $$u=e_{x}Ae^{-\alpha_{1}x}e^{i(\omega t-kx+\theta )}$$

(10) $$\alpha =\alpha_{e}+\alpha_{1}=\beta /v_{p}+\alpha_{1}=R_{m}/2mv_{p}+\alpha_{1}$$

Equation (10) is the acoustic attenuation coefficient.

Equation (10) ensures that, under the continuous steady-state sine external force, any particle in any spatial position will, after undergoing a transient process from rest to steady-state sine vibration, vibrate according to the sine pattern, that is, enter the steady-state sine vibration state, and its amplitude will no longer change with time. However, for particles distributed at different spatial positions in a viscoelastic medium, their amplitudes will decrease exponentially with the increase in the propagation distance of the acoustic wave. In other words, the amplitude of the steady-state sine acoustic wave propagating in a viscoelastic solid decreases only with the increase in the propagation distance.

The acoustic attenuation coefficient described by Equation (10) takes into account both the energy loss caused by the damping of particle vibration and the energy loss resulting from the wave propagation attenuation. The mass of the particle is equal to the product of its volume and the density of the medium, and the frictional force is proportional to the viscosity of the medium. Under the condition that the geometric parameters of the particle remain unchanged, Equation (10) indicates that: (i) The acoustic attenuation coefficient can be decomposed into two independent components: one is the propagation attenuation caused by wave propagation in the spatial domain, and the other is the vibration attenuation resulting from particle vibration in the time domain. (ii) The vibration attenuation of particles only depends on the physical properties of the viscoelastic solid itself and the geometric size of the particles, and it is independent of the external excitation conditions. (iii) The propagation attenuation is not only affected by the above physical properties and geometric parameters, but also closely related to the frequency of the sine acoustic wave, showing obvious frequency-dependent characteristics. The greater the additional propagation attenuation (i.e., particle vibration damping), the greater the impact on the amplitude of the steady-state sine acoustic wave propagating in the viscoelastic medium; conversely, the impact will be reduced.

### 2.3. The Electric–Acoustic and Acoustic–Electric Conversion

Let us discuss the electric–acoustic (for acoustic source) and acoustic–electric (for receiver) conversion characteristics of the transducers.

**Figure 1:**
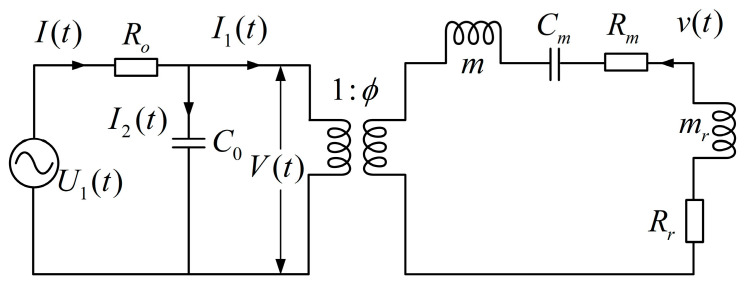
Electric–mechanical equivalent circuit of a thin-disk source transducer.

The equivalent circuits for the electric–acoustic conversion of the acoustic source transducer are shown in Figure 1 [20,21]:

From Figure 1, the impulse function, system function, and oscillation frequency of the acoustic source transducer that characterize the electric–acoustic conversion process can be expressed by

(11) $$h_{1}(t)=A_{3}e^{-\alpha t}\varepsilon (t)+B_{3}e^{-\beta t}\cos(\omega_{1s}t+\varphi_{1})\varepsilon (t)$$

(12) $$H_{1}(i\omega )={\left.H_{1}(s)\right|}_{s=i\omega }=\frac{i\omega d}{-i\omega^{3}-\omega^{2}a+i\omega b+c}$$

(13) $$\omega_{1s}=\sqrt{3}B$$

where $a=\frac{1}{C_{0}R_{0}}+\frac{R_{m}+R_{r}}{m+m_{r}}$, $b=\left(\frac{R_{m}+R_{r}}{C_{0}R_{0}}+\frac{1}{C_{m}}+\frac{\phi^{2}}{C_{0}}\right)/(m+m_{r})$, $c=\frac{1}{(m+m_{r})C_{0}C_{m}R_{0}}$, $d=\frac{\phi }{(m+m_{r})C_{0}R_{0}}$, $\phi =\pi r_{0}^{2}h_{33}/\left(\beta_{33}^{3}l_{t}\right)$ (mechanical–electrical conversion coefficient), $\alpha_{0}=a/3-2A$, $\beta_{0}=a/3+A$, $A=\left(x+y\right)/2$, $B=\left(x-y\right)/2$, $x={\left(-q+\sqrt{D}\right)}^{1/3}$, $y={\left(-q-\sqrt{D}\right)}^{1/3}$, $D=p^{3}+q^{2}={(b/3+a^{2}/9)}^{3}+{(a^{3}/27-ab/6+c/2)}^{2}$, $A_{3}=\frac{-d\alpha_{0}}{\sigma^{2}+3B^{2}}$, $B_{3}=2\sqrt{M^{2}+N^{2}}$, $\sigma =\beta -\alpha_{0}$, $\varphi_{1}=\arctan(N/M)$, $N=-\frac{d(\beta \sigma +3B^{2})}{\sqrt{3}B(\sigma^{2}+3B^{2})}$, $M=\frac{d\alpha }{\sigma^{2}+3B^{2}}$.

The above characteristics reflect the conversion relationship between the voltage driving signal (i.e., the excitation) applied to the acoustic source transducer and its surface vibration velocity. It should be noted that the input of the acoustic source transducer is the voltage driving signal (excitation), and the output is the vibration velocity of the transducer’s surface (response). The system function is the mathematical expression that describes the transducer’s electric–acoustic conversion characteristic from voltage excitation to surface vibration velocity.

**Figure 2:**
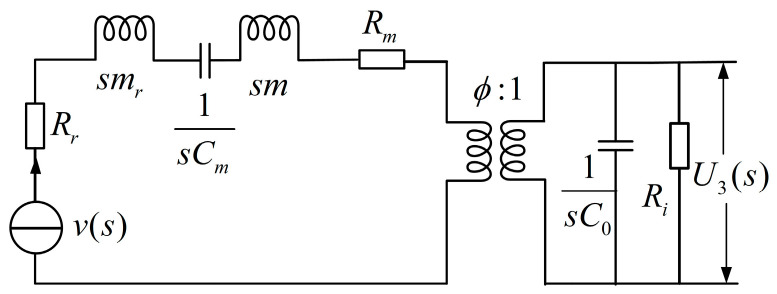
Electric–mechanical equivalent circuit of a thin-disk receiving transducer.

Similarly, the equivalent circuits for the acoustic–electric conversion of the receiving transducer are shown in Figure 2.

The impulse function, system function, and oscillation frequency that characterize the acoustic–electric conversion process, describing how the particle displacement velocity (excitation signal) acting on the receiving transducer is converted into a voltage signal, can be written as:

(14) $$h_{3}(t)={A_{3}}^{\prime }e^{-\alpha^{\prime }t}\varepsilon (t)+{B_{3}}^{\prime }e^{-\beta^{\prime }t}\cos(\omega_{3s}t+\varphi_{3})\varepsilon (t)$$

(15) $$H_{3}(i\omega )=\frac{i\omega d^{\prime }}{-i\omega^{3}-\omega^{2}a^{\prime }+i\omega b^{\prime }+c^{\prime }}$$

(16) $$\omega_{3s}=\sqrt{3}B^{\prime }$$

where $a^{\prime }=\frac{1}{C_{0}R_{i}}+\frac{R_{m}+R_{r}}{m+m_{r}}$, $b^{\prime }=\frac{1}{m+m_{r}}\left(\frac{R_{m}+R_{r}}{C_{0}R_{i}}+\frac{1}{C_{m}}+\frac{\phi^{2}}{C_{0}}\right)$, $d^{\prime }=\frac{\rho_{0}C_{0}\phi }{(m+m_{r})C_{0}}$, $c^{\prime }=\frac{1}{(m+m_{r})C_{0}R_{i}}\left(1/C_{m}+\phi^{2}/C_{0}\right)$, $p^{\prime }=b^{\prime }/3+{a^{\prime }}^{2}/9$, $q^{\prime }={a^{\prime }}^{3}/27-a^{\prime }b^{\prime }/6+c^{\prime }/2$, $D^{\prime }={p^{\prime }}^{3}+{q^{\prime }}^{2}$, $x^{\prime }=\sqrt[{3}]{-q^{\prime }+\sqrt{D^{\prime }}}$, $y^{\prime }=\sqrt[{3}]{-q^{\prime }-\sqrt{D^{\prime }}}$, $A^{\prime }=\left(x^{\prime }+y^{\prime }\right)/2$, $B^{\prime }=\left(x^{\prime }-y^{\prime }\right)/2$, $\alpha^{\prime }=a^{\prime }/3-2A^{\prime }$, $\beta^{\prime }=a^{\prime }/3+A^{\prime }$, $\sigma^{\prime }=\beta^{\prime }-\alpha^{\prime }$, ${A_{3}}^{\prime }=\frac{-d^{\prime }\alpha^{\prime }}{{\sigma^{\prime }}^{2}+3{B^{\prime }}^{2}}$, ${B_{3}}^{\prime }=2\sqrt{{M^{\prime }}^{2}+{N^{\prime }}^{2}}$, $M^{\prime }=\frac{d^{\prime }\alpha^{\prime }}{{\sigma^{\prime }}^{2}+3{B^{\prime }}^{2}}$, $N^{\prime }=-\frac{d^{\prime }(\beta^{\prime }\sigma^{\prime }+3{B^{\prime }}^{2})}{\sqrt{3}B^{\prime }({\sigma^{\prime }}^{2}+3{B^{\prime }}^{2})}$, $\varphi_{3}=\arctan\left(N^{\prime }/M^{\prime }\right)$.

The equation for calculating the time delay caused by the electric–acoustic/acoustic–electric conversions of the acoustic source/receiving transducers can be written as:

(17) $$\Delta t_{l}=\frac{2\pi \varphi_{l}(\omega_{i})}{360^{\circ }\omega_{i}}$$

where 360° is the angle corresponding to one complete cycle of a sine signal with angular frequency *ω*; the phase shift angle $\varphi_{l}(\omega_{i})$ is a function of frequency, with the subscripts {*l*} = {1, 3} corresponding to the phase shift of the acoustic source transducer during the electric–acoustic conversion process, and the phase shift of the receiving transducer during the acoustic–electric conversion process, respectively.

## 3. Results and Discussion

### 3.1. Acoustic Attenuation

Plexiglas, T-sandstone, and Shale are selected as the propagation media for simulation and analysis. Table 1 lists the relevant physical parameters of plexiglas, T-sandstone, and Shale [22].

Plexiglas is an artificial homogeneous material, whereas T-sandstone and Shale, as typical natural rock media, possess complex internal structures. When acoustic waves propagate through rocks, in addition to viscous dissipation within the solid, energy is lost due to pore structures and microcracks. Therefore, the equivalent viscosity coefficient of rocks is generally significantly higher than that of artificial homogeneous materials. Based on this difference, to ensure that the model results not only reflect the true attenuation characteristics of the media but also remain interpretable, the viscosity coefficient for each medium is selected from its reasonable parameter range. Specifically, the viscosity coefficient $\eta_{11}$ of plexiglas is selected as $5.0\times 10^{-4}$ $N\cdot {s/m}^{2}$, $1.0\times 10^{-3}$ $N\cdot {s/m}^{2}$, $1.5\times 10^{-3}$ $N\cdot {s/m}^{2}$, and $2.0\times 10^{-3}$ $N\cdot {s/m}^{2}$, respectively; the viscosity coefficients $\eta_{11}$ of T-sandstone and Shale are selected as $2.0\times 10^{-2}$ $N\cdot {s/m}^{2}$, $5.0\times 10^{-2}$ $N\cdot {s/m}^{2}$, $1.0\times 10^{-1}$ $N\cdot {s/m}^{2}$, and $2.0\times 10^{-1}$ $N\cdot {s/m}^{2}$, respectively, to study the influence of different viscosity parameters on the acoustic attenuation characteristics of plexiglas and the two rock media.

The acoustic attenuation coefficients of different viscoelastic media differ significantly in amplitude. If the maximum values of each are used directly for normalization, it will weaken the contrast in attenuation intensities across different media. Therefore, in this paper, the plexiglas and the rock are normalized separately.

**Figure 3:**
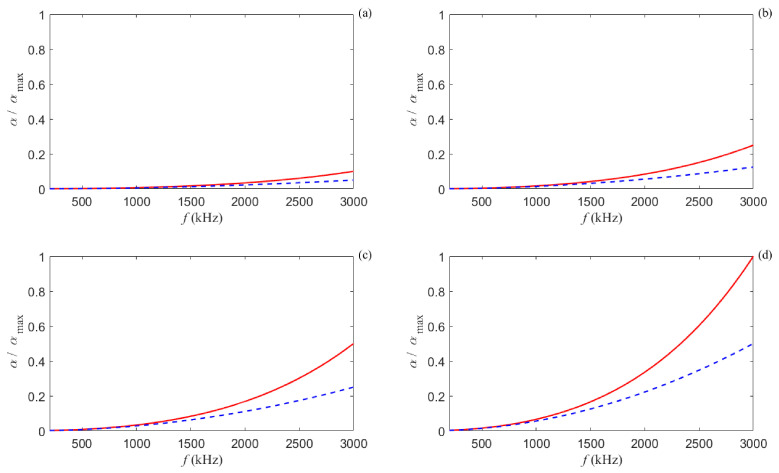
The relationship between *α* and *f* for plexiglas with different viscosity coefficients: (**a**) $\eta_{11}=2.0\times 10^{-2}$ $N\cdot {s/m}^{2}$, (**b**) $\eta_{11}=5.0\times 10^{-2}$ $N\cdot {s/m}^{2}$; (**c**) $\eta_{11}=1.0\times 10^{-1}$ $N\cdot {s/m}^{2}$; (**d**) $\eta_{11}=2.0\times 10^{-1}$ $N\cdot {s/m}^{2}$.

**Figure 4:**
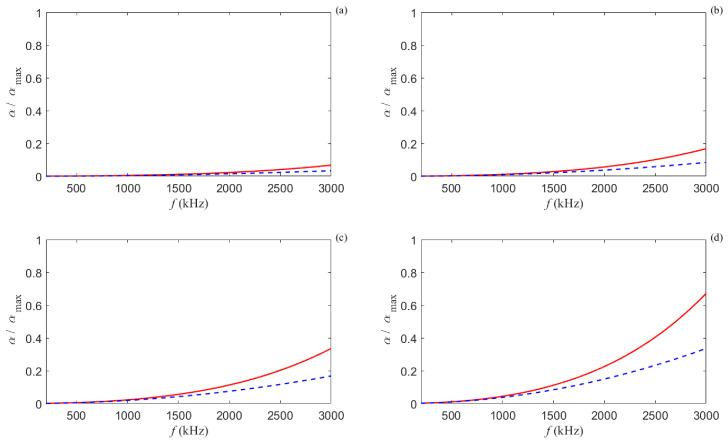
The relationship between *α* and *f* for T-sandstone with different viscosity coefficients: (**a**) $\eta_{11}=2.0\times 10^{-2}$ $N\cdot {s/m}^{2}$, (**b**) $\eta_{11}=5.0\times 10^{-2}$ $N\cdot {s/m}^{2}$; (**c**) $\eta_{11}=1.0\times 10^{-1}$ $N\cdot {s/m}^{2}$; (**d**) $\eta_{11}=2.0\times 10^{-1}$ $N\cdot {s/m}^{2}$.

**Figure 5:**
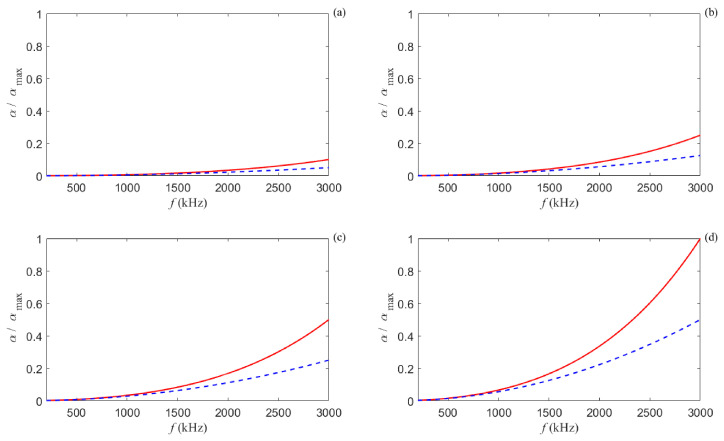
The relationship between *α* and *f* for Shale with different viscosity coefficients (**a**) $\eta_{11}=2.0\times 10^{-2}$ $N\cdot {s/m}^{2}$, (**b**) $\eta_{11}=5.0\times 10^{-2}$ $N\cdot {s/m}^{2}$, (**c**) $\eta_{11}=1.0\times 10^{-1}$ $N\cdot {s/m}^{2}$, (**d**) $\eta_{11}=2.0\times 10^{-1}$ $N\cdot {s/m}^{2}$.

Based on the above parameter settings, using Equations (5) and (10), respectively, the relationships between the acoustic attenuation coefficient and frequency for different viscosity coefficients were calculated, as shown in Figure 3, Figure 4 and Figure 5. Among them, for plexiglass, the maximum acoustic attenuation coefficient corresponding to its maximum viscosity coefficient is used to normalize the calculation results, as shown in Figure 3; for T-sandstone and Shale, the maximum attenuation coefficient corresponding to the maximum viscosity coefficient of Shale is used to normalize the calculation results of the two types of rocks, as shown in Figure 4 and Figure 5. It should be noted here that in Figure 3, Figure 4 and Figure 5, *α* represents the acoustic attenuation coefficient for two cases of considering damping attenuation and not considering damping attenuation, and *α*_max_ represents the maximum value of the acoustic attenuation coefficient in the above two cases; the dashed blue–line is the normalized curve of the acoustic attenuation coefficient versus frequency for only considering the attenuation of wave propagation [i.e., *α*_1_ in Equation (5)]. In contrast, the solid red–line is the normalized curve of the acoustic attenuation coefficient versus frequency, accounting for both wave-propagation attenuation and particle-vibration damping [i.e., α in Equation (10)].

The calculation results in Figure 3, Figure 4 and Figure 5 indicate: (i) The solid line values (from the proposed model) are always greater than the dashed line values (from the Auld model), therefore, for acoustic waves propagating in viscoelastic solids, the amplitude attenuation is jointly determined by wave propagation attenuation and particle vibration damping. (ii) The larger the viscosity coefficient of the medium, the larger the acoustic attenuation coefficients for the above two models. (iii) The attenuation coefficients in both models increase with frequency. (iv) In the low-frequency range, the difference between the attenuation coefficients from the two models has some minor change with frequency, but it is not obvious. (v) In the high-frequency region, the difference between the two models increases rapidly with the increase in frequency.

### 3.2. Electric–Acoustic/Acoustic–Electric Conversion Characteristics of Piezoelectric Transducers

**Figure 6:**
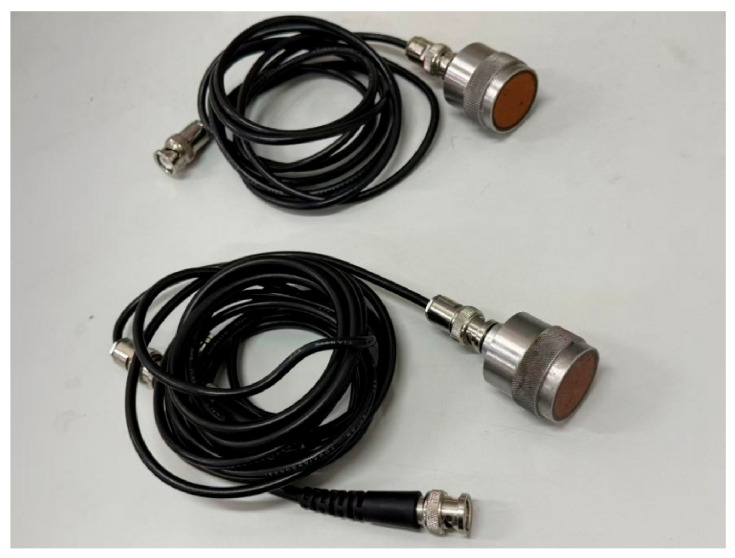
Physical images of the source and receiving transducers.

The acoustic source and receiving piezoelectric disk transducers used in this study are identical. Both are made of the piezoelectric material PZT, with a radius of 1.5 cm and a thickness of 0.15 cm, and are polarized in the thickness direction, as shown in Figure 6. The relevant physical parameters of the piezoelectric material PZT are listed in Table 2.

**Figure 7:**
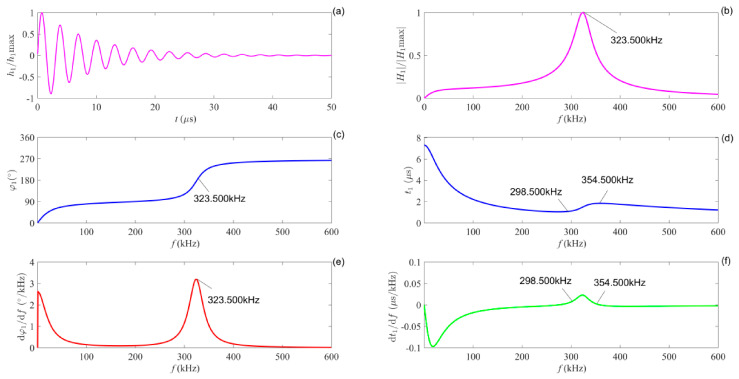
Electric-acoustic conversion characteristics of a thin-disk transducer: (**a**) impulse response; (**b**) amplitude spectrum; (**c**) phase spectrum; (**d**) time delay; (**e**) derivative of phase with respect to frequency; (**f**) derivative of time delay with respect to frequency.

**Figure 8:**
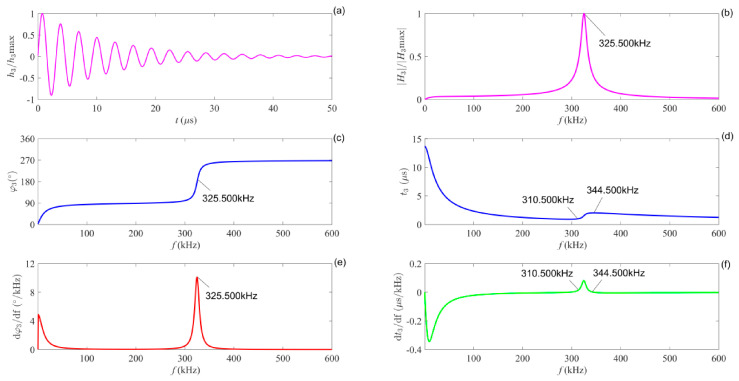
Acoustic-electric conversion characteristics of the thin-disc transducer: (**a**) impulse response; (**b**) amplitude spectrum; (**c**) phase spectrum; (**d**) time delay; (**e**) derivative of phase angle with respect to frequency; (**f**) derivative of time delay with respect to frequency.

According to Equations (11)–(17), as well as the geometric dimensions, physical parameters, and piezoelectric constants of the thickness-polarized disk transducer shown in Table 2, the calculated electric–acoustic impulse response, amplitude spectrum, phase spectrum, generated time delay, derivative of phase shift with respect to frequency, and derivative of time delay with respect to frequency for the acoustic source transducer, as well as the acoustic–electric impulse response, amplitude spectrum, phase spectrum, generated time delay, derivative of phase shift with respect to frequency, and derivative of time delay with respect to frequency for the receiving transducer, are shown in Figure 7 and Figure 8, respectively.

The calculation results in Figure 7 and Figure 8 show: (i) When the transducer acts as an acoustic source, the center frequency is $f_{10}$ = 123.500 kHz, and when it acts as a receiver, the center frequency is $f_{30}$ = 325.500 kHz. The former has a slightly lower center frequency than the latter. (ii) Regardless of whether the transducer is as the acoustic source or a receiver, the phase shifts $\varphi_{1}$ and $\varphi_{3}$ produced by it change with the frequency *f* in a basically consistent pattern, and the time delays *t*_1_ and *t*_3_ also change with the frequency *f* in a basically consistent pattern. (iii) Within all frequency ranges, the derivatives d$\varphi_{1}$/d*f* and d$\varphi_{3}$/d*f* of the phase shift with respect to frequency are both greater than zero, so the phase shifts $\varphi_{1}$ and $\varphi_{3}$ increase monotonically with the increase of frequency *f*. (iv) In most frequency ranges, the derivatives d*t*_1_/d*f* and d*t*_3_/d*f* of the time delay with respect to frequency are negative values. The time delays *t*_1_ and *t*_3_ decrease with the increase of frequency f, indicating that the phase shift and the time delay change with frequency show an opposite relationship. (v) However, in the regions near the center frequencies of the transducer, that is, within the frequency range of 298.500–354.500 KHz for the acoustic source transducer and within the frequency range of 310.500–344.500 KHz for the receiving transducer, the derivatives d*t*_1_/d*f* and d*t*_3_/d*f* of the time delay change to positive values. At this time, the time delays *t*_1_ and *t*_3_ show a positive relationship with the phase shift as a function of frequency; that is, as frequency increases, both the phase shift and the time delays increase.

## 4. Acoustic Attenuation Measurement Methods and Experimental Validation

**Figure 9:**
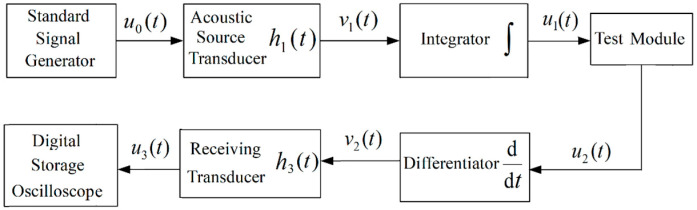
Block diagram of the experimental measurement setup.

The block diagram of the experimental measurement setup is shown in Figure 9. The system consists of a standard signal generator, an acoustic source transducer, a receiving transducer, and a digital storage oscilloscope. A plexiglas cylinder was used as the test module. As illustrated in Figure 6, the two transducers were placed in close contact with the two cylindrical cross-sections of the test module. The standard signal generator outputs a constant-amplitude sine-wave voltage, which excites the acoustic source transducer. Following a transient transition process, the vibration generated by the acoustic source transducer radiates a steady-state sine acoustic wave outward. Similarly, all particles in the test module reach a steady-state sine-vibration state after the transient transition. Once the sine wave signals have passed through the test module and the entire measurement system (including the receiving transducer) has reached steady-state sine vibration, the acoustic waves are converted into steady-state sine electrical signals by the receiving transducer. These signals are then recorded and displayed using a digital storage oscilloscope. During the experiment, the frequency of the sine excitation voltage signal output by the standard signal generator is gradually varied while the amplitude is kept constant.

When the entire system reaches a stable sine-vibration state, the electrical output signal from the receiving transducer is measured, and both the amplitude of the steady-state sine signal and its corresponding frequency are recorded. By sequentially repeating this measurement process, the relationships of both the amplitude and phase for the measured steady-state sine signal versus the frequency are obtained—that is, the electrical signal output from the electrical terminals of the receiving transducer (i.e., the measured acoustic signal).

In the figure, let the steady-state sine excitation voltage signal $u_{0}(t)$ output by the TFG6025A have an angular frequency; $v_{1}(t)$ and $u_{1}(t)$ are the vibration velocity and displacement of all particles inside the acoustic source transducer; $u_{2}(t)$ and $v_{2}(t)$ are the displacement and vibration velocity of the particles at the receiving transducer location after all particles inside the test module have reached steady-state sine vibration following the acoustic wave emission from the source; $u_{3}(t)$ is the steady-state sine voltage signal output from the electrical terminal of the receiving transducer. Furthermore, we have the parameters of the electric–acoustic conversion impulse response ($h_{1}(t)$) of the acoustic source transducer and the acoustic–electric conversion impulse response ($h_{3}(t)$) of the receiving transducer.

A sine signal with an amplitude of *A* and an angular frequency of *ω*, after passing through a system with a system function of *H*(*jω*), its output remains a sine periodic signal of the same frequency, with only the amplitude and phase changing, which can be expressed as:

(18) y(t) = A|H(jω)|sin(ωt+φ)

φ=atanIm{H(jω)}Re{H(jω)}

Let the acoustic source transducer be located at the origin of the coordinate system.

During the measurement process, we applied sine excitation voltage signals with a constant amplitude of *A* to the acoustic source transducer at a fixed frequency interval and conducted *N* measurements. According to Formula (18) and the acoustic measurement process shown in Figure 9, the sine excitation voltage signal corresponding to the *i*th measurement (*i* = 1, 2, 3, …, *N*) can be expressed as:

(19) $$u_{0i}(t)\;=\;A\sin\left(\omega_{i}t\right)$$

Then, the steady-state sine vibration velocity at the surface of the acoustic source transducer can be expressed as:

(20) $$v_{1i}(t)\;=\;A|H_{1}(j\omega_{i})|\sin(\omega_{i}t+\varphi_{1i})$$

where $H_{1}(j\omega_{i})$ and φ1i=atanIm{H1(jωi)}Re{H1(jωi)} are the electric–acoustic conversion function and the phase shift angle corresponding to $h_{1}(t)$ at the frequency $f_{i}=\omega_{i}/2\pi$.

The vibration velocity of the acoustic source transducer, as given by Equation (20), can be converted to the corresponding displacement by integration.

(21) $$\begin{array}{cc} u_{1i}(t) & =\int v_{1i}(t)dt\\ & =\int A|H_{1}(j\omega_{i})|\sin(\omega_{i}t+\varphi_{1i})dt\\ & =-\frac{A|H_{1}(j\omega_{i})|}{\omega_{i}}\cos(\omega_{i}t+\varphi_{1i}) \end{array}$$

Suppose a sine acoustic wave with frequency $f_{i}$ propagates from the acoustic source transducer located at the coordinate origin to the observation point (i.e., the spatial position of the receiving transducer), with the distance between them being *x*. Combining the real part of Formula (9) with Formula (21), the displacement of the vibrating particle at the observation point *x* can be expressed as:

(22) $$u_{2i}(x,t)=-\frac{A|H_{1}(j\omega_{i})|}{\omega_{i}}e^{-\alpha_{i}x}\cos(\omega_{i}t-k_{i}x+\varphi_{1i})$$

Because the input signal (excitation) at the mechanical end of the receiving transducer is the particle displacement velocity, it is differentiated by a differentiator to obtain the displacement particle velocity, i.e.,

(23) $$v_{2i}(x,t)=\frac{du_{2i}(x,t)}{dt}=A|H_{1}(j\omega_{i})|e^{-\alpha_{i}x}\sin(\omega_{i}t-k_{i}x+\varphi_{1i})$$

The particle displacement velocity $v_{2i}(x,t)$ is used as the excitation signal and is input to the mechanical end of the receiving transducer. After it reaches the steady-state sine working state, the signal output from the electric end of the transducer remains a steady-state sine voltage signal (that is, the measured steady-state sine acoustic wave signal), which can be expressed as:

(24) $$u_{3i}(x,t)==A|H_{1}(j\omega_{i})||H_{3}(j\omega_{i})|e^{-\alpha_{i}x}\sin(\omega_{i}t-k_{i}x+\varphi_{1i}+\varphi_{3i})$$

During the measurement process, the source distance (the distance between the acoustic source and the receiver) *x* is fixed, with time *t* as the variable. The amplitude of the steady-state sine voltage signal is measured. Therefore, according to Formula (24), we can obtain:

(25) $$e^{-\alpha_{i}x}=\frac{u_{3i}(x,t{)|}_{\max}}{A|H_{1}(j\omega_{i})||H_{3}(j\omega_{i})|}$$

Taking the logarithm of both sides yields the acoustic attenuation coefficient of the sine acoustic wave with angular frequency $\omega_{i}$ as follows:

(26) $$\alpha_{i}=-\frac{1}{x}\ln\frac{u_{3i}(x,t{)|}_{\max}}{A|H_{1}(j\omega_{i})||H_{3}(j\omega_{i})|}$$

We keep the amplitude of the sine excitation voltage signal constant and continuously change its frequency step by step, measure the amplitude spectrum of the sine acoustic wave signal (electrical signal) output from the electrical end of the receiving transducer, and then, according to Equation (26), we can invert the relationship between the acoustic attenuation of the propagation medium (i.e., viscoelastic solid) and the frequency.

In the experimental measurement, a plexiglas cylinder with a length of 260 mm and a diameter of 120 mm was selected as the test module, and the measurement frequency range was from 100 kHz to 510 kHz. A sine voltage excitation signal with constant amplitude (peak-to-peak value of 20 V) was applied to the acoustic source transducer by increasing the frequency in steps of 1 kHz. Each time the acoustic source transducer was excited with a sine-voltage signal at a given frequency, the entire system reached a steady-state sine-vibration condition, and the measurement was performed.

**Figure 10:**
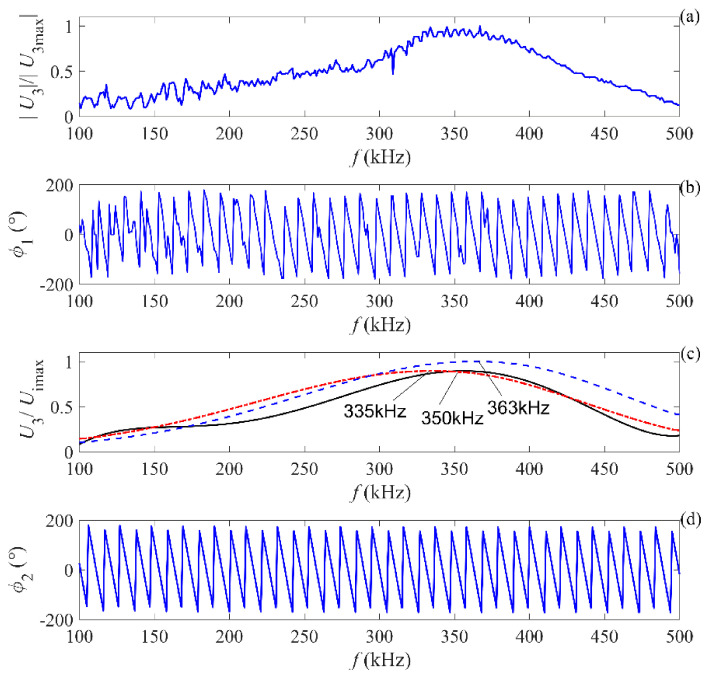
Relationships of both the amplitude and phase shift of the steady-state sine voltage signal at the electrical end of the receiving transducer versus the frequency: (**a**) measured amplitude–frequency characteristic; (**b**) measured phase–frequency characteristic; (**c**) comparison of amplitude–frequency characteristics; (**d**) theoretical calculated phase–frequency characteristic.

Figure 10a,b present the experimentally measured amplitude and phase spectra, respectively, of the electrical output voltage signal from the receiving transducer.

Figure 10c compares three normalized amplitude spectra: the blue dashed-curve corresponds to the theoretically predicted spectrum accounting solely for propagation attenuation (ignoring particle vibration damping), with a central frequency of 363 kHz; the red dotted-curve represents the theoretical spectrum incorporating both propagation attenuation and particle vibration damping, yielding a central frequency of 335 kHz; and the black solid-curve is an empirical fit to the measured amplitude spectrum shown in Figure 10a, exhibiting a central frequency of 350 kHz. All three curves in Figure 10c are normalized to the peak amplitude of the undamped theoretical spectrum (i.e., the blue dashed curve). Figure 10d displays the theoretically derived phase spectrum of the electrical output voltage signal, computed under the damping-inclusive model.

Figure 10 shows that the center frequency of the electrical output signal’s amplitude spectrum from the receiving transducer varies significantly under different conditions. When considering damping attenuation, the theoretically calculated value and the experimentally measured fitting value are 335 kHz and 350 kHz, respectively; whereas without damping attenuation, the theoretical value is 363 kHz. It can be seen that the latter is significantly higher than the former, indicating that damping attenuation has a significant pulling-down effect on the center frequency.

**Figure 11:**
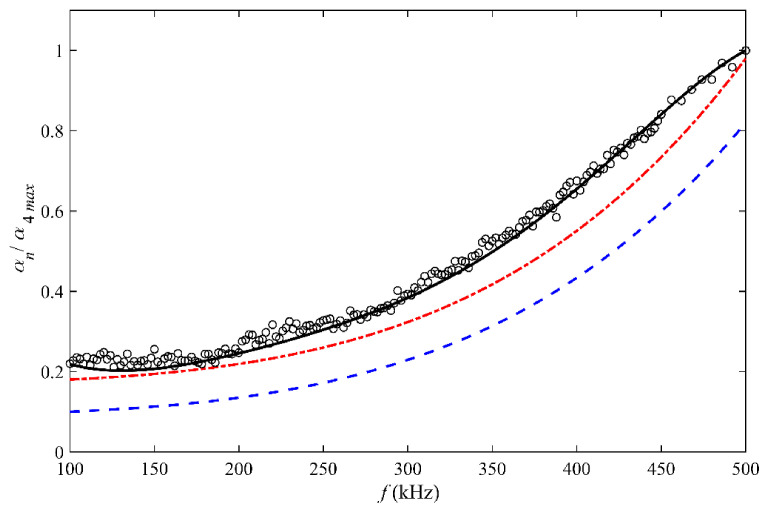
Frequency dependence of acoustic attenuation coefficient: comparison of inversion values with theoretical values.

Based on Formula (26) and the amplitude and phase spectra shown in Figure 10, the relationships between the inverted acoustic attenuation coefficients and frequency are depicted in Figure 11. Where *α_n_* represents the acoustic attenuation coefficient; the subscript {*n*} = {1, 2} represents the relationship curve between the acoustic attenuation coefficient and frequency calculated theoretically without considering damping attenuation (blue dashed-line) and that with considering damping attenuation (red dotted line); the subscript {*n*} = {3, 4} represents the relationship curve of the acoustic attenuation coefficient versus frequency obtained by inversion using measurement data (small circles) and that fitted by using measurement data (black solid line); $\alpha_{4\max}$ represents the maximum value of the acoustic attenuation coefficient after fitting processing.

In this study, measurements and comparisons were conducted within the frequency band range of 100 kHz to 500 kHz. It was mainly because when the frequency deviated from this range, the amplitude of the steady-state sine electrical signal measured at the electrical end of the receiving transducer significantly decreased, and it might even be drowned out by background noise, making it difficult to reliably acquire the effective signal.

In the experimental measurement and theoretical calculation, a total of *N* = 398 frequency points were selected for data collection. Firstly, using the measured voltage signal from the electrical end of the receiving transducer, the acoustic attenuation coefficient is inversely calculated and then fitted. Taking the fitted acoustic attenuation coefficient as the reference value, the average errors between the theoretical acoustic attenuation and the measured fitted acoustic attenuation coefficient were calculated in two cases: with damping attenuation considered and without damping attenuation considered.

The results show that the average error when damping attenuation is considered is

(27) $${\mathrm{ME}}_{1}=\frac{1}{N}\sum_{i=1}^{N}|\alpha_{f}(\omega_{i})-\alpha (\omega_{i})|\times 100\%=5.91\%$$

and the average error without considering damping attenuation is

(28) $${\mathrm{ME}}_{2}=\frac{1}{N}\sum_{i=1}^{N}|\alpha_{f}(\omega_{i})-\alpha_{1}(\omega_{i})|\times 100\%=16.18\%$$

Here, $\alpha_{f}(\omega_{i})$ represents the value of the fitted acoustic attenuation coefficient at the angular frequency $\omega =\omega_{i}$; $\alpha (\omega_{i})$ is the value of the acoustic attenuation coefficient considering damping attenuation at the angular frequency $\omega =\omega_{i}$; $\alpha_{1}(\omega_{i})$ is the value of the acoustic attenuation coefficient without considering damping attenuation at the angular frequency $\omega =\omega_{i}$.

The calculation results show that, compared with the experimental measurement values, the error in the acoustic attenuation coefficient obtained by the theoretical calculation that considers damping attenuation is significantly smaller than that obtained without considering damping attenuation. Furthermore, both theoretical calculations and experimental inversion results, accounting for wave-propagation attenuation and particle-vibration damping, indicate that the acoustic attenuation coefficients obtained, whether predicted by theoretical models or inverted from measured data, are greater than those derived theoretically when only propagation attenuation is considered.

## 5. Conclusions and Future Work

This work presented an improved analytical expression for the acoustic attenuation coefficient, incorporating the damping mechanism of particle vibration within the medium and the attenuation mechanism of wave propagation. It provides a new theoretical framework for describing acoustic attenuation in viscoelastic media.

Considering the inertial effect of particles in viscoelastic solid media (i.e., the mass effect), a new method for inverting the acoustic attenuation coefficient from measurement data is proposed, effectively eliminating the influence of characteristics introduced during the electrical–acoustic and acoustic–electrical conversion processes of the acoustic source transducer and the receiving transducer, thereby improving the objectivity and reliability of the inversion results.

A new method is proposed to invert the acoustic attenuation coefficient from measured data, eliminating the influence of the electric–acoustic/acoustic–electric conversion characteristics of the acoustic source/receiving transducer.

Theoretical calculations and inversion results based on experimental data show that the obtained acoustic attenuation coefficients are significantly greater than the traditional theoretical values that account only for propagation attenuation, indicating that the physical mechanism by which particle vibrations and wave propagation jointly determine the attenuation of acoustic waves propagating in viscoelastic media.

In follow-up studies, we plan to develop a series of test modules encompassing a range of geometric dimensions and medium types. Meanwhile, both the sound source and the receiver will be discretized into multiple small unit sources and unit receivers. From a vector analysis perspective, we will systematically investigate the contributions of each unit to projections and the effective superposition of units under varying spatial positions and orientations. This approach is intended to provide a more comprehensive characterization of the acoustic field distribution in practical application scenarios and to further enhance the accuracy of inferring the acoustic wave attenuation coefficient from measured data.

## Figures and Tables

**Table 1:** Physical parameters of plexiglas, T-sandstone, and Shale. T-sandstone is an abbreviation for Taylor sandstone.

**Medium**	$c_{11}$ (N/m)	$v_{p}$ (m/s)	ρ (kg/m^3^)
plexiglas	$8.80\times 10^{9}$	2730	1180
T-sandstone	$2.84\times 10^{10}$	3368	2500
shale	$1.76\times 10^{10}$	2745	2340

**Table 2:** Physical parameters of piezoelectric material PZT.

**Physical Parameters**	$c_{33}^{D}$ (N/m^2^)	$h_{33}$ (V/m)	$\varepsilon_{33}^{S}/\varepsilon_{0}$	ρ (kg/m^3^)
Numerical value	$1.52\times 10^{11}$	$1.69\times 10^{9}$	1450	$6\times 10^{3}$

## Data Availability

The original contributions presented in this study are included in the article. Further inquiries can be directed to the corresponding authors.
